# Genomic Analysis of Highly Virulent Georgia 2007/1 Isolate of African Swine Fever Virus

**DOI:** 10.3201/eid1704.101283

**Published:** 2011-04

**Authors:** David A.G. Chapman, Alistair C. Darby, Melissa Da Silva, Chris Upton, Alan D. Radford, Linda K. Dixon

**Affiliations:** Author affiliations: Institute for Animal Health, Woking, UK (D.A.G. Chapman, L.K. Dixon);; University of Liverpool, Neston, UK (A.C. Darby, A. Radford);; University of Victoria, Victoria, British Columbia, Canada (M. Da Silva, C. Upton)

**Keywords:** African swine fever, viruses, genomic analysis, virulence, outbreak, Republic of Georgia, expedited, research

## Abstract

African swine fever is widespread in Africa but has occasionally been introduced into other continents. In June 2007, African swine fever was isolated in the Caucasus Region of the Republic of Georgia and subsequently in neighboring countries (Armenia, Azerbaijan, and 9 states of the Russian Federation). Previous data for sequencing of 3 genes indicated that the Georgia 2007/1 isolate is closely related to isolates of genotype II, which has been identified in Mozambique, Madagascar, and Zambia. We report the complete genomic coding sequence of the Georgia 2007/1 isolate and comparison with other isolates. A genome sequence of 189,344 bp encoding 166 open reading frames (ORFs) was obtained. Phylogeny based on concatenated sequences of 125 conserved ORFs showed that this isolate clustered most closely with the Mkuzi 1979 isolate. Some ORFs clustered differently, suggesting that recombination may have occurred. Results provide a baseline for monitoring genomic changes in this virus.

## Abstract 2

Sequence information will facilitate research on vaccine development.

African swine fever (ASF) is a hemorrhagic fever in domestic pigs that causes serious economic losses and high mortality rates. ASF is currently endemic to many countries in sub-Saharan Africa and the island of Sardinia in Europe and was endemic to Spain and Portugal from 1960 until the mid 1990s. It is still endemic to Madagascar since its introduction in 1998. Sporadic ASF outbreaks have occurred in Brazil, the Caribbean region, the Indian Ocean island of Mauritius, and countries in Europe (*1*). There is no vaccine against ASF, and disease control relies on rapid diagnosis and implementation of quarantine and slaughter policies. African swine fever virus (ASFV) is a large, icosahedral, cytoplasmic, double-stranded DNA virus; it is the only member of the family *Asfaviridae*, although it shares similarities with other virus families in the superfamily of nucleo-cytoplasmic large DNA viruses (*2**–**4*).

In 2007, a new outbreak of ASF was confirmed in the Republic of Georgia, which is far from the usual geographic virus range in sub-Saharan Africa. Infections were first observed near the Black Sea port of Poti and are thought to have been introduced by improper disposal of waste from shipping. The disease rapidly spread throughout Georgia and was reported in Armenia and in wild boar in Chechnya in the Russian Federation in 2007 and Azerbaijan in 2008. ASF has since spread to 9 regions in the Russian Federation, including 2,000 km to St. Petersburg in October 2009. As of August 10, 2010, there have been 85 outbreaks reported within the Russian Federation, which have led to the deaths of ≈48,000 animals and an estimated cost to the Russian economy during 2009 of US$1 billion (*5*; World Organisation for Animal Health Information Database). There have been several reports of ASFV infection in wild boars in different locations in the Russian Federation, which led to fears that ASF may have become established in the wild boar population. This rapid transboundary spread of ASF emphasizes the serious risk for ASF to pig farming worldwide.

In its natural hosts (warthogs [*Phacochoerus aethiopicus*], bushpigs [*Potamochoerus porcus*], and *Ornithodorous* spp. soft ticks), ASFV causes a persistent but asymptomatic infection. In domestic swine, it causes an acute hemorrhagic infection with mortality rates <100%. European wild boars (*Sus scrofa*) are susceptible, and disease signs are similar to those in domestic pigs. The ASFV strain introduced to the Caucasus is highly virulent and resulted in a mortality rate of ≈100% during the early stages of the outbreak in Georgia; ≈90,000 animals died or were destroyed (http://web.oie.int/wahis/public.php?page=home). Experimental infections of pigs confirmed that isolates obtained after introduction of ASF into Armenia and the Russian Federation cause acute disease and result in high mortality rates (www.efsa.europa.eu/en/scdocs/scdoc/1556.htm).

Genotyping of ASFV isolates by partial sequencing of the B646L gene that encodes the major capsid protein p72 has identified 22 genotypes (*6*). The Georgia 2007/1 isolate was grouped within genotype II by partial sequencing of the B646L and B602L genes and complete sequencing of the CP204L gene. Genotype II virus has been isolated in Mozambique and Zambia and was also introduced into the Indian Ocean islands of Madagascar (1998) and Mauritius (2007) (*7*).

We analyzed the complete coding region of the genome of the Georgia 2007/1 strain of ASFV, which was isolated after its introduction to Georgia in 2007. This information provides a baseline for comparison with other isolates obtained during the continued spread of ASF in this region and provides information for vaccine and diagnostic test development.

## Methods

### Viruses and Cells

The Georgia 2007/1 isolate was obtained from tissue samples from pigs submitted to the World Organisation for Animal Health Reference Laboratory at the Institute for Animal Health, Pirbright, UK, on June 4, 2007 (*7*). Primary porcine bone marrow cells cultured in Earle saline media at a concentration of 4 × 10^6^ cells/mL were infected with virus at a multiplicity of infection of 1. Virus-containing cell supernatants were collected 4 days postinfection. Virus-containing cell supernatant was used for purification of virus DNA.

### Purification of Virus DNA

Virus supernatant was centrifuged at 118,000 × *g* (SW 32 Ti Rotor; Beckman Coulter, Brea, CA, USA) for 1 h at 4°C. Pelleted virus was resuspended in RSB buffer (10 mmol/L NaCl, 10 mmol/L Tris-HCl, 1 mmol/L EDTA) containing 0.01 M MgCl_2_ and DNase I (Sigma, St. Louis, MO, USA) (200 μg/mL) and incubated for 1 h at 37°C to digest contaminating cellular DNA. EDTA (50 mmol/L) was then added to inactivate DNase. Virus was then centrifuged through a 20% sucrose RSB cushion at 62,000 × *g* (70.1 Ti Rotor; Beckman Coulter) for 95 min at 4*°*C. Virus pellets were resuspended in 1 mL of buffer (10 mmol/L Tris-HCl, 1 mmol/L EDTA). RNase (40 µg/mL), proteinase K (200 µg/mL), and sodium dodecyl sulfate (1% final concentration) were added, and samples were incubated for 18 h at 37°C. Viral genomic DNA was extracted with phenol and precipitated with ethanol. To remove low molecular weight nucleic acid, viral DNA was further purified by using the Elu-Quick Kit (Whatman, Maidstone, UK) according to the manufacturer’s protocol II.

### Sequence Determination and Analysis

DNA for sequencing was amplified from 100 ng of purified viral DNA by using the Repli-G Kit (QIAGEN, Valencia, CA, USA). This method uses an isothermal multiple displacement amplification and a processive DNA polymerase capable of replicating <100 kbp. The DNA polymerase has a 3′ → 5′ exonuclease proofreading activity to maintain high fidelity in the amplified products. Nucleotide sequence of the complete coding regions of the genome of the Georgia 2007/1 isolate was determined by using a Roche (Basel, Switzerland) 454 GS FLX sequencer. Analysis of genome sequences, open reading frames (ORFs), and orthologous protein families were conducted by using Artemis (*8*), Glimmer software (*9*) and programs available at Viral Bioinformatics–Canada (*10**,**11*). ORFs were compared with the related ASFV genome sequences (Mkuzi 1979 isolate, GenBank accession no. AY261362 and Genotype I, Benin 97/1 isolate, GenBank accession no. AM712239) to identify potential frame shifts in the genome that interrupted reading frames. Regions of uncertainty were sequenced by PCR amplification of fragments and Sanger sequencing to confirm the sequence. These uncertainties were located mainly in homopolymer sequences, which have been reported to cause ambiguities during Roche 454 sequencing (*12**,**13*). The GenBank accession no. for the genome sequence is FR682468.

## Results

### Sequence of Coding Regions

The final assembly of the Georgia 2007/1 isolate produced a genome of 189,344 bp, not including terminal inverted repeats and cross links. This genome is considerably larger than genomes of attenuated ASFV isolates BA71V (GenBank accession no. NC_001659) (170,101 bp) and OURT88/3 (GenBank accession no. AM712240) (171,719 bp). In contrast, genomes available for virulent isolates range from 182,284 bp to 193,886 bp. Dot-plot comparisons of the Georgia 2007/1 genome with other genomes showed that these genomes were collinear, although deletions or insertions were observed in the regions close to the genome termini, particularly in the left genome end as in genomes of other isolates. Most size differences result from gain or loss of members of 5 multigene families (MGF 100, MGF 110, MGF 300, MGF 360, and MGF 530) (*14**–**16*).

### Genomic Analysis

Using GATU software (*10*), we identified 166 ORFs (Technical Appendix). Of these ORFs, 125 are present in all 11 ASFV isolates sequenced to date. The conserved ORFs include those that encode for structural proteins; proteins involved in virus assembly, enzymes and other factors involved in nucleotide metabolism, DNA replication and repair, mRNA transcription and processing; several involved in regulating host cell pathways; 16 members of the MGFs; and several of unknown function. Of the remaining 42 ORFs, which are not conserved between all 11 ASFV isolates sequenced, 24 are members of the 5 MGFs. The GATU software identified ORFs on the basis of those encoded in reference genomes. To determine if other ORFs may be present, we used Glimmer software (*9*). This analysis identified 189 ORFs, the additional 23, all encoded proteins of <64 aa that lacked sequence similarity with known proteins (Technical Appendix). Eleven of these ORFs overlapped or were entirely within other larger ORFs. Thus, these ORFs are not likely to represent functional genes.

### Genome Comparison of the Georgia 2007/1 Isolate with other ASFV Isolates

**Figure 1:**
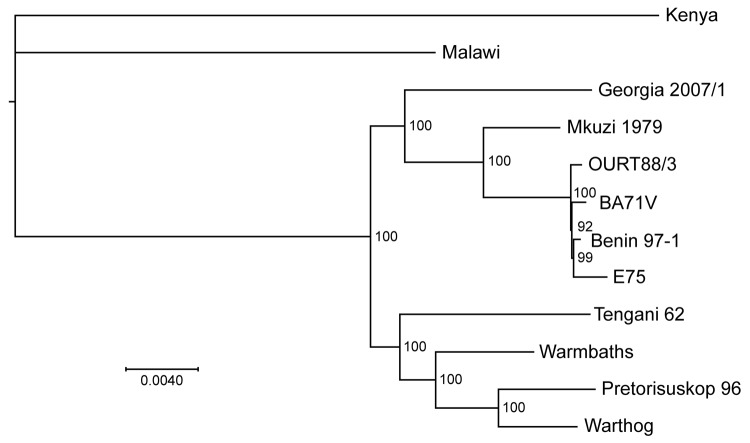
Comparison of the Georgia 2007/1 African swine fever virus (ASFV) isolate genome with those of other ASFV isolates. ASFV phylogeny midpoint was rooted in a neighbor-joining tree on the basis of 125 conserved open reading frame regions (40,810 aa) from 12 taxa. Node values show percentage bootstrap support (n = 1,000). The isolates shown and accession numbers are Kenya AY261360, Malawi Lil20/1 AY261361, Tengani AY261364, Warmbaths AY261365, Pretorisuskop AY261363, Warthog AY261366, Warmbaths AY261365, Mkuzi AY261362, OurT88/3 a.m.712240, BA71V NC_001659, Benin97/1 a.m.712239, and E75 FN557520. Scale bar indicates nucleotide substitutions per site.

To determine the phylogenetic relationship between the Georgia 2007/1 isolate and other ASFV isolates (Table), we compared the concatenated amino acid sequences of proteins encoded by 125 conserved ORFs comprising 40,810 aa (Figure 1). This phylogenic analysis shows that most isolates cluster in 2 main clades. The first group comprises isolates from West Africa and Europe belonging to genotype I. The Mkuzi 1979 and Georgia 2007/1 isolate also fall within this group but are more distantly related to genotype I isolates. The second group comprises other isolates from eastern and southern Africa (Tengani 62, Warthog, Warmbaths, Pretoriskup 96). Two isolates, Malwai lil 20/1 and Kenya 1950, are outliers from these groups.

**Table:** Characteristics of African swine fever virus isolates analyzed

Isolate	Country	Host	Year	Virulence	GenBank accession no.	Reference
Georgia 2007/1	Georgia	Domestic pig	2007	High	FR682468	This study
BA71qqV	Spain	Domestic pig	1971	Tissue culture adapted	U18466	(*15*)
Benin 97/1	Benin	Domestic pig	1997	High	AM712239	(*14*)
OURT 88/3	Portugal	Tick	1988	Low	AM712240	(*17*)
Kenya	Kenya	Domestic pig	1950	High	AY261360	(*18*)
Malawi Lil20/1	Malawi	Tick	1983	High	AY261361	(*19*)
Mkuzi	Zululand	Tick	1978	Unknown	AY261362	(*18*)
Pretorisuskop/96/4	South Africa	Tick	1996	High	AY261363	(*18*)
Tengani 62	Malawi	Domestic pig	1962	High	AY261364	(*20*)
Warmbaths	South Africa	Tick	1987	Unknown	AY261365	(*18*)
Warthog	Namibia	Warthog	1980	Unknown	AY261366	(*18*)

Comparison of complete genomes shows that most variation is at the left end of the genomes and is caused by presence or absence of different numbers of members of the MGFs (*14**,**15*). Some ORF deletions are observed close to the right genome end, notably between the tissue culture–adapted BA71V isolate and other isolates, including Georgia 2007/1. The isolates showing greatest sequence divergence from the Georgia 2007/1 isolate are the Kenyan 1950 and Malawi Lil20/1 isolates. This sequence divergence is greatest toward the left end of the genome.

A total of 78 ORFs in all 11 isolates share >90% aa identity of proteins encoded. Of these ORFs, only 33 have a confirmed or predicted function. The most conserved proteins include the histone-like structural protein A104R, which is 99%–100% identical in all isolates. The bcl-2-bax homologue (A179L) protein has 98.9%–100% aa identity in all isolates except Kenya 1950 and Benin 97/1, which are 94% identical compared with that of the Georgia 2007/1 isolate and other isolates. Several of the other most conserved proteins encoded are enzymes, including helicase A859L (>95% identity), RNA helicase B962L (>95% identity), prenyltransferase B318L (>95% identity), RNA polymerase 6 C147L (>96% identity), and DNA primase C962R (>97% identity).

The more divergent proteins include several with immunomodulatory functions, such as A238L, which varies in amino acid identity from 58.9% (Malawi Lil20/1) to 81.3% (Mkuzi) compared with Georgia 2007/1. The C-type lectin-like protein EP153R shows 54.9% (Warmbaths) to 79.7% (Warthog) aa identity compared with Georgia 2007/1. The CD2v protein encoded by the EP402R ORF varies from 65.8% (Tengani isolate) to 86.1% (Malawi Lil20/1 genotype VIII). The CD2v and EP153R proteins are transmembrane proteins with reported roles in evading host defenses (*21**,**22*). The thymidylate kinase (A240L) protein is divergent whereas most other ORFs that encode enzymes are highly conserved. The C84L and E66Lproteins of unknown function also have variable sequences. The virulence-associated protein DP71L (*23**–**25*) is encoded by ASFV isolates as 1 of 2 forms differing in size. Only genotype VIII isolates from Malawi and Zambia and the Kenya isolates encode the long form. All other isolates, including Georgia 2007/1, encode the short form.

The structural protein P22 (*26*), encoded by the KP177R ORF, is present in only 1 copy near the left genome end of the BA71V isolate; this ORF is present in all the other isolates. However, in the other isolates, there are either 1 or 2 additional ORFs related to KP177R near the right end of the genome. The Georgia 2007/1 isolate contains 1 copy (l10L) of the KP177R-related ORF, close to the right end of the genome. The amino acid identity between the KP177R protein and the related proteins is low, e.g., the 2 proteins share only 42.2% aa identity in the Georgia 2007/1 isolate. Much higher amino acid identity is shared between the proteins encoded by orthologous ORFs from different isolates. For example, the P22 protein is greater than 78% identical across all the genomes analyzed.

**Figure 2:**
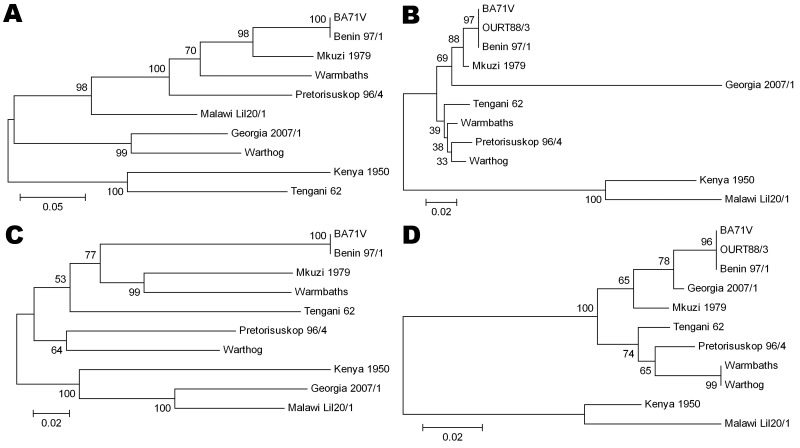
Phylogenetic trees of 4 of the most divergent African swine fever virus proteins. A) C-type lectin EP153R, B) A238L, C) CD2-like protein EP402R, D) structural protein K177R (P22). Evolutionary history was inferred by using the neighbor-joining method. The bootstrap consensus tree inferred from 1,000 replicates is taken to represent the evolutionary history of the proteins analyzed. Branches corresponding to partitions reproduced in <50% bootstrap replicates are collapsed. The percentage of replicate trees in which the associated proteins clustered in the bootstrap test (1,000 replicates) are shown next to the branches. The tree is drawn to scale, with branch lengths in the same units as those of the evolutionary distances used to infer the phylogenetic tree. All positions containing gaps and missing data were eliminated from the dataset (complete deletion option). There were 224 positions in the final dataset. Phylogenetic analyses were conducted in MEGA4 (*27*). Scale bars indicate amino acid substitutions per site.

Phylogenetic analysis was conducted for proteins encoded by each ORF. Although most proteins showed the same clustering as observed for that of the concatenated conserved ORFs (Figure 1) for several proteins, the Georgia isolate sequence clustered differently. Examples of phylogenetic trees for proteins encoded by 4 ORFs are shown in Figure 2. The A238L and KP177R protein sequences from the Georgia 2007/1 isolate cluster the same as the concatenated conserved 125 proteins, the EP402R protein sequence from the Georgia 2007/1 sequence clusters more closely with the Malawi Lil20/1 and Kenya 1950 isolates, and the EP153R protein sequence from the Georgia 2007/1 isolate clustered more closely with the Warthog isolate. A possible explanation for these observations is that recombination may have occurred. If so, we might expect to find several adjacent ORFs that cluster in the same way and differently from the conserved concatenated ORFs. One such example is observed with the adjacent ORFs l7L (100%), l8L (100%), l9R (100%), and l10L (91.2%) from the Georgia 2007/1 isolate, which encode proteins with the highest amino acid identity with the genotype XIX Warthog isolate. Analysis across the 125 concatenated conserved protein sequences clusters the Georgia 2007/1 isolate more closely with Mkuzi 1979 isolate. However, there is no clear evidence for recent recombination events.

### Multigene Families

The presence or absence of some members of MGF 360 and MGF 505/530 families correlates closely with pathogenesis in ASFV, and the complement of these present in the Georgia 2007/1 isolate is as expected for a highly pathogenic isolate. The nonpathogenic isolate OURT 88/3 and the tissue culture–adapted isolate BA71V have deletions of 5 or 6 members, respectively, of MGF 360 and 2 or 1 members, respectively, of MGF 530 (*14*) that are in all other pathogenic isolates sequenced, including the Georgia 2007/1 isolate. Deletion of these members of the MGF 360 and MGF 530 families from the genome of the pathogenic Pretoriuskop 96/4 isolate dramatically reduced virus virulence in domestic pigs (*28*).

The Georgia 2007/1 isolate has 37 members of the different MGFs (Technical Appendix). In addition, the Georgia 2007/1 isolate only has 1 member of MGF 100 (MGF100–1R) in comparison with other genomes, which have 2 or 3 MGF 100 members. The Georgia 2007/1 genome contains 12 of the 14 known members of MGF 110, including a fusion of MGF 110 5L and 6L (MGF 110–5L/6L). The fusion of these 2 ORFs was confirmed by Sanger sequencing to ensure that it was not a sequencing error. The Georgia 2007/1 isolate has only 2 of the 4 members of MGF 300 compared with a minimum of 3 of 4 found in all other genomes. This isolate contains 15 members of MGF 360; the number present in the other genomes varies from a minimum of 11 or 12 in the BA71V and OURT88/3 isolates, respectively, to 18 in the Kenyan isolate of the 22 MGF genes identified. MGF 505/530 appears to be closely conserved across most genomes. The Georgian2007/1 isolate and 6 other isolates (Benin97/1, Mkuzi, Pretorisuskop, Tengani, Warmbaths, and Warthog) contain 10 of the 11 MGF 505/530 members identified. Nonpathogenic isolates OURT88/3 and BA71V lack 2 (MGF 505/530–1R and –2R) or 1 (MGF 505/530–1R) of the MGF 505/530 ORFs, respectively. Further investigation into the role of individual members of the 5 MGFs on interferon response is ongoing.

## Discussion

The continuing outbreak of ASF in the Caucasus region is caused by a highly virulent strain of ASFV that belongs to genotype II (*7*). Comparison of the nucleotide sequence of the genome of the Georgian 2007/1 isolate with other isolates indicated that it is most closely related to isolate Mkuzi 1979 (Figure 1). The Mkuzi 1979 isolate was obtained from a tick isolate in Zululand near Mozambique where genotype II isolates have been found in domestic pigs. Phylogenetic analysis of concatenated protein sequences from 125 conserved ORFs results in clustering of the Georgia 2007/1 and Mkuzi 1979 isolates with genotype I isolates, although more divergent than other members of this group from West Africa and Europe (Benin 97/1, OURT88/3, BA71V and E75) and other isolates from eastern and southern Africa, including Tengani, 62, Warthog, Warmbaths, and Pretorisuskop 96/4. The eastern Africa isolates Malawi Lil20/1 and Kenya 1950 form a separate and more distantly related cluster.

Analysis of the phylogeny of individual proteins (Figure 2) does not always match the clustering observed by comparison of concatenated conserved ORFs (Figure 1). A possible explanation for this observation is that recombination events have occurred. These observations indicate that caution should be used when inferring phylogenetic relationships between ASFV isolates based on a small number of genes. ASFV isolates have been grouped into 22 genotypes by partial sequencing of the ORF encoding the p72 major capsid protein B646L. However, analysis of the protein sequence encoded by this ORF does not reflect the phylogeny, as indicated by analysis of the concatenated conserved ORFs and of other individual ORFs. Complete genome sequence analysis provides the most information; as viral genome analysis and sequencing becomes more routine, this procedure will become the method of choice. In the short term, targeted sequence analysis of several ORFs, including those that more closely cluster with that of the concatenated conserved ORF sequences, will provide a more accurate estimate of phylogenetic relationships rather than analysis of 1 ORF such as B646L.

Comparison of the rates of synonomous versus nonsynonomous substitutions across ASFV genes identified 14 or 18 genes that are undergoing positive selection (*29*). These genes included 2 of the proteins (CD2v and EP153R) that we identified as being most divergent at the amino acid level.

Determination of the sequence of the ASFV isolate that was introduced into the Caucasus region provides a benchmark to which other isolates from this epidemic can be compared. This finding may enable sequence changes to be related to any changes in phenotype of the virus. In addition, detailed knowledge of the sequence will facilitate research on vaccine development by enabling the genes encoded to be expressed and assayed for their ability to confer protection in pigs. It will also facilitate the design of rationally attenuated vaccines by sequential deletion of genes involved in immune evasion and virulence.

## Supplementary Material

Technical Appendix Open reading frames of African swine fever virus.
